# Association Between White Matter Microstructure and Verbal Fluency in Patients With Multiple Sclerosis

**DOI:** 10.3389/fpsyg.2019.01607

**Published:** 2019-07-18

**Authors:** Tal Blecher, Shmuel Miron, Galit Grimberg Schneider, Anat Achiron, Michal Ben-Shachar

**Affiliations:** ^1^Gonda Multidisciplinary Brain Research Center, Bar-Ilan University, Ramat Gan, Israel; ^2^Multiple Sclerosis Center, Sheba Medical Center, Tel Hashomer, Israel; ^3^Sackler School of Medicine, Tel Aviv University, Tel Aviv, Israel; ^4^Department of English Literature and Linguistics, Bar-Ilan University, Ramat Gan, Israel

**Keywords:** diffusion MRI, tractography, language pathways, letter-based fluency, category-based fluency, multiple sclerosis

## Abstract

Verbal fluency refers to the ability to generate words quickly and efficiently according to predefined phonological or semantic criteria. Deficits in verbal fluency limit patients’ ability to communicate effectively and to function well in social setups. Multiple sclerosis (MS) patients suffer from various cognitive impairments, and some of them experience language deficits as well. The goal of this study is to examine the contribution of the dorsal and ventral language pathways to verbal fluency in MS patients. All patients (*N* = 33) underwent diffusion MRI (dMRI) and fluency measurements. Diffusion parameters were calculated along dorsal and ventral language-related pathways and their right-hemispheric homologs, identified individually in each patient. Significant correlations were found between fluency measures and mean fractional anisotropy (FA) in several pathways, including the left fronto-temporal arcuate fasciculus (AF_ft_), bilateral inferior fronto-occipital fasciculus (IFOF), and bilateral frontal aslant tract. Along-tract correlations revealed a more selective pattern of associations: letter-based fluency was associated with FA in a segment of the left AF_ft_ (dorsal pathway), while category-based fluency was associated with FA in a segment of the right IFOF (ventral pathway). The observed pattern of associations, mapping letter-based fluency to the dorsal stream and category-based fluency to the ventral stream, fits well within the dual stream framework of language processing. Further studies will be necessary to assess whether these associations generalize to the typical adult population or whether they are tied to the clinical state.

## Introduction

Verbal fluency concerns our ability to access and produce words quickly and efficiently, which is an essential prerequisite for effective communication and social functioning. Tests of verbal fluency require search, access, selection, retrieval and pronunciation of as many words as possible in a restricted time period, based on a predefined criterion. This process may fail due to deficits in any of these cognitive components (Thompson-Schill et al., 1998; Troyer et al., 1998; Lacey et al., 2017). Fluency tasks are also considered effective probes for executive function, and are commonly included in neuropsychological batteries that assess such executive skills (e.g., Kramer et al., 2014). Performance on fluency tasks is impaired in numerous clinical conditions, including aphasia, focal epilepsy and Alzheimer’s disease (Baldo et al., 2010; Metternich et al., 2014; Rodríguez-Aranda et al., 2016). Therefore, assessment of verbal fluency is conducted as part of the clinical protocol in many clinical populations with acute and degenerative conditions, including patients with multiple sclerosis, who are the focus of this study (Friend et al., 1999; Benedict et al., 2006; Grimberg Schneider, 2014; Gerstenecker et al., 2017).

Multiple sclerosis (MS) is a chronic, autoimmune inflammatory disease of the central nervous system (CNS) (Casanova et al., 2003). The pathology of MS is characterized by demyelination, axonal damage, gliosis, and multifocal lesions (i.e., plaques) in both white and gray matter of the CNS. MS affects many cognitive abilities, including attention, memory, and executive function (Foong et al., 1997; Rovaris and Filippi, 2000; Zakzanis, 2000; Nocentini et al., 2001; Achiron and Barak, 2003, 2006). However, the evidence for language impairments, particularly in the early stages of MS, is inconclusive (Henry and Beatty, 2006; Rogers et al., 2007; Chiaravalloti and Deluca, 2008). Some studies report that language function is relatively preserved in MS patients, primarily of the relapsing-remitting subtype (D’Esposito et al., 1996), while others demonstrate a substantial impairment in verbal fluency and naming abilities in all MS subtypes (Heaton et al., 1985; Achiron et al., 1992; Friend et al., 1999; Drake et al., 2002; Huijbregts et al., 2004; Henry and Beatty, 2006; Negreiros et al., 2008; Cáceres et al., 2011; Abad et al., 2015; Geisseler et al., 2016; Gerstenecker et al., 2017). One prospective study found no deficit in verbal fluency in the early stages (first 5 years) of MS (Grimberg Schneider, 2014). Since MS patients demonstrate considerable variability in language performance and in quantitative measures of white matter microstructure, they constitute a relevant clinical population for studies of neurocognitive associations. This study focuses on the associations between microstructural properties of white matter and different components of verbal fluency performance.

Verbal fluency is typically assessed using two standard tasks: Letter-based (phonemic) fluency and category-based (semantic) fluency (Abwender et al., 2001; Henry and Crawford, 2004). In both tasks, participants are requested to generate and pronounce as many words as possible in 1 min, according to a predefined criterion. This criterion may be an opening letter (letter-based fluency) or a semantic category (category-based fluency) (Borzowski et al., 1967; Kavé and Knafo-Noam, 2015). Overall, individuals’ category-based fluency is typically higher than their letter-based fluency. The developmental trajectory is similar for fluency components: they both show a positive, rising slope until the age of 20 years, and begin to decline after the age of 40 years (Sauzeon et al., 2004; Chávez-Oliveros et al., 2015; Friesen et al., 2015; Kavé and Knafo-Noam, 2015). It is noteworthy that the decline in category-based fluency scores is much steeper compared to that of letter-based fluency (Brickman et al., 2005). Differences between the two types of fluency may arise from different levels of practice or difficulty involved in each process, as well as from the differential efficiency of distinct cerebral pathways that may be involved in those processes.

Functional magnetic resonance imaging (fMRI) studies of verbal fluency show that fluency tasks activate a left-lateralized network of cortical regions (Birn et al., 2010). This left lateralization changes along the lifespan and decays with aging (Meinzer et al., 2009; Nagels et al., 2012; Peters et al., 2012). Other studies which investigated the different components of verbal fluency suggested that the right hemisphere has a larger involvement in processing category-based fluency compared to letter-based fluency (Schlösser et al., 1998; Donnelly et al., 2011; Kircher et al., 2011; Nagels et al., 2012; Shapira-Lichter et al., 2013; Glikmann-Johnston et al., 2015). Both letter-based fluency and category-based fluency engage many cortical regions along the known language pathways, including the left inferior frontal gyrus (IFG), anterior cingulate, left temporal regions, superior parietal cortex, left hippocampus thalamus, and cerebellum (Phelps et al., 1997; Gourovitch et al., 2000; Abrahams et al., 2003; Costafreda et al., 2006; Robinson et al., 2012; Biesbroek et al., 2016). Only a few fMRI studies compared the two fluency tasks directly (Mummery et al., 1996; Gourovitch et al., 2000; Costafreda et al., 2006; Birn et al., 2010). In those cases, letter-based fluency was associated with dorsal regions of the left IFG, whereas category-based fluency was associated with ventral regions of the left IFG (Costafreda et al., 2006; Birn et al., 2010). In addition, superior parietal cortex and occipito-temporal cortices (bilaterally) were involved in letter-based fluency, while the left fusiform gyrus and the early visual cortices were involved in category-based fluency (Birn et al., 2010). Others suggested that left frontal regions respond more strongly to letter-based fluency while left temporal regions respond more strongly to category-based fluency (Mummery et al., 1996; Gourovitch et al., 2000). Lesion studies support this dissociation, showing that patients with frontal lesions sustained more severe letter-based fluency deficits, while they could still generate many examples for semantic categories. On the other hand, patients with temporal lesions had category-based fluency deficits but were able to generate words according to phonological constraints (Baldo et al., 2006, 2010). A recent finding demonstrated that lesions in the right IFG are also associated with a category-based fluency deficit (Biesbroek et al., 2016). Taken together, the findings so far support the involvement of multiple, distant cortical regions in both fluency components, with some differences in relative contribution of the frontal and temporal regions to each fluency component, and potentially some involvement of the right hemisphere in addition to the classical language network.

Frontal and temporal language regions are connected through a set of dorsal and ventral white matter pathways (Saur et al., 2008). Functional dissociations have been established between these pathways, in agreement with the dual-stream model of speech processing (Hickok and Poeppel, 2007). According to this model, the dorsal stream maps speech sounds to production and engages in phonological processing of speech, while the ventral stream extracts semantic information essential for speech understanding (Hickok and Poeppel, 2004, 2007; Saur et al., 2008). Recent diffusion magnetic resonance imaging (dMRI) studies in healthy adults revealed a positive association between verbal fluency and structural properties of the left arcuate fasciculus, a key dorsal pathway (Peters et al., 2012; Allendorfer et al., 2016). Much of the information about verbal fluency and white matter comes from studies in patients suffering from stroke and degenerative aphasia, Alzheimer’s disease or epilepsy. A TBSS study in patients with Alzheimer’s disease demonstrated an association between both fluency components and fractional anisotropy (FA) within voxels of the left superior longitudinal fasciculus (SLF, dorsal) and the left inferior fronto-occipital fasciculus (IFOF, ventral), along with the corpus callosum. Letter-based fluency was uniquely associated with FA in other voxels of the left SLF, while category-based fluency was associated with FA in voxels of the right IFOF and right SLF (Rodríguez-Aranda et al., 2016). In patients with primary progressive aphasia and in adults who stutter correlations were reported between verbal fluency and microstructural measures of the frontal aslant tract, connecting the IFG and ventral precentral gyrus with the supplementary and pre-supplementary motor areas (Catani et al., 2013; Kinoshita et al., 2015; see also Kronfeld-Duenias et al., 2016 for a related finding).

In the current paper, we examined the association between white matter microstructure and verbal fluency in a sample of 33 patients with MS. In each patient, we identified dorsal and ventral language-related pathways, bilaterally, and quantified diffusivity properties along the extent of the tract. We hypothesized that significant associations with verbal fluency would be detected in left dorsal pathways, due to the articulatory component of this task. In addition, we hypothesized that bilateral ventral pathways will show associations with verbal fluency, particularly category based, due to its involvement in semantic access. Testing these predictions in MS patients is of interest because it will further elucidate the involvement of the language pathways in this disease.

## Materials and Methods

### Participants

Participants included 33 patients with multiple sclerosis (MS), selected for the current study out of a larger group of 78 patients included in a previous study (Grimberg Schneider, 2014, see below for selection and exclusion criteria). All patients were recruited at the MS Center at Sheba Medical Center (Tel Hashomer, Israel), and diagnosed with relapsing-remitting, primary or secondary progressive MS. All participants were native Hebrew speakers. Patients were selected for the current study if they had undergone a diffusion MRI (dMRI) scan within a year from the cognitive testing session (this inclusion criterion did not result in any systematic bias in terms of participants’ age, disease duration or fluency measures, see Supplementary Table S1). Exclusion criteria included acute MS relapse or severe dysarthria (which can impair fluency). Disability was assessed using the expanded disability status scale (EDSS, Kurtzke, 1983). This scale includes evaluation of several functional systems, such as motor, sensory, visual, mental, cerebellar and more. An experienced neurologist graded the patients’ functioning in each domain, from 0 = no disability to 5 or 6 = maximal disability, based on a physical examination and their disease history. Finally, a total integrated score was calculated for each patient (between 0 = normal examination and 10 = death due to MS). The data were collected as part of the routine follow up of the patients. Participants signed a written informed consent in accordance with the Declaration of Helsinki. Participants indicated that they agree that their data may be used anonymously for research purposes. The study was approved by the ethics committee of Sheba Medical Center.

### Behavioral Data Acquisition

Verbal fluency was assessed using the standard procedure described in Kavé (2005) for Hebrew [see The Delis-Kaplan Executive Function System (Delis et al., 2001) for the original English version of this test]. The verbal fluency test is divided into two parts: letter-based fluency and category-based fluency. In both parts, patients were provided with a cue (an opening letter or a semantic category), and were asked to provide as many Hebrew words as possible, within 60 s, according to the cue. Each aspect of verbal fluency was sampled using three different cues, 60 s long each. In letter-based fluency, patients were asked to produce words that begin with the letter *Bet* (/b/), *Gimel* (/g/), and *Shin* (/sh/). In category-based fluency, patients were asked to produce words that belong to the category *animals, vehicles*, and *fruits and vegetables*. For detailed instructions see Kavé (2005).

Additional cognitive testing was conducted using “MindStreams,” a computerized global assessment battery (NeuroTrax Corporation, Bellaire, TX, United States) (Dwolatzky et al., 2003). This cognitive assessment was performed by a trained examiner at the MS center at Sheba Medical Center. We used the executive function and attention scores from this battery. The executive function score was composed of three subtests: the go/no-go response inhibition test, the Stroop interference test, and the catch game test. The go/no-go response inhibition test was a timed continuous performance test. Patients were instructed to respond to large colored stimuli of any color, except when the stimulus is red, in which case no response was to be made. In the Stroop interference test, patients performed a two alternative forced choice version of the classic Stroop task. Finally, in the catch game test, patients were asked to “catch” (click on) a rectangular object that falls vertically from the top of the screen. A standardized composite score of executive function was calculated automatically by the MindStreams software, based on patients’ age and education and their performance on the three subtests mentioned so far.

The attention score was further calculated based on the go – no go response inhibition test, the Stroop interference test, and an information processing test. In the information processing test, participants made a numerical judgment (≤4 or >4) for single-digit stimuli, two digit calculations (e.g., 5-1) or three digit calculations (e.g., 3+1-2). Stimuli were presented at a slow, medium or fast speed. A standardized composite score of attention was calculated automatically by the MindStreams software, based on patients’ age and education and their performance on these three subtests.

### MRI Data Acquisition

MRI data were collected using a 3.0T scanner (Signa Excite, General Electric Medical Systems, Milwaukee, WI, United States) located at the Sheba Medical Center (Tel Hashomer, Israel). Scanning was conducted with an eight-channel head coil for parallel imaging. Head motion was minimized by padding the head with cushions, and patients were asked to stay still during the scan.

A standard dMRI protocol was applied by means of a single-shot, spin-echo, diffusion-weighted, echo-planar imaging sequence (∼60 axial, 2.6 mm thick slices, no gap; FOV = 256 mm × 256 mm, matrix size = 256 × 256, providing a voxel size of 1 mm × 1 mm × 2.6 mm). Diffusion-weighted volumes were acquired along 31 non-collinear directions (*b* = 1000 s/mm^2^) and two reference volumes (*b* = 0 s/mm^2^). The scan volume was adjusted to cover the entire brain in each patient, so the exact number of slices varied slightly between patients. Total scan time for the dMRI sequence was ∼8 min. High resolution T1-weighted anatomical images were acquired for each patient using a 3D fast spoiled gradient-recalled echo sequence (FSPGR; 155 ± 11 axial slices, slice thickness = 1 mm, covering the entire cerebrum; voxel size: 1 mm × 1 mm × 1 mm).

### Data Analysis

#### Behavioral Data Analysis

For each patient, age-standardized scores were calculated for letter-based fluency and for category-based fluency. To do so, we first calculated the total number of words produced for the three letter cues (letter-based sum) and for the three category cues (category-based sum). We then converted these sums into age-standardized *Z*-scores, using published norms for adult Hebrew speakers (Kavé, 2005).

Prior to this calculation, fluency data were preprocessed as follows: Errors and repetitions were excluded. When two homonyms were provided, the second mention was counted only if the patient pointed out the alternate meaning explicitly. Words inflected in both masculine and feminine forms were counted as one. Synonyms were counted as separate words. In category-based fluency, names of subcategories (e.g., a bird) were not counted if specific items within that subcategory (e.g., dove, eagle) were also provided. Slang terms, as well as foreign words, were generally considered acceptable (Kavé, 2005).

#### Imaging Data Analysis

##### Data preprocessing

Data preprocessing was conducted using the open sourced “mrDiffusion” package^1^ and MATLAB 2012b (The Mathworks, Natick, MA, United States). Preprocessing followed the same standard steps as in our previous publications (Dougherty et al., 2007; Yeatman et al., 2011; Blecher et al., 2016; Kronfeld-Duenias et al., 2016), as detailed below.

First, T1 images were aligned to an ac-pc orientation: the locations of the anterior and posterior commissures were identified manually on the T1 of each patient and these points were used to align the anatomical T1 volume to a canonical ac–pc orientation, using a rigid body transformation (no warping was applied). Second, distortions in the diffusion-weighted images due to eddy currents and subject motion were corrected by a 14-parameter constrained non-linear co-registration algorithm based on the expected pattern of eddy-current distortions (Rohde et al., 2004). Third, diffusion images were registered to the ac-pc aligned T1 anatomical images. Alignment was achieved by registering the b0 images to the resampled T1 image using a rigid-body, mutual information maximization algorithm (implemented in SPM5; Friston and Ashburner, 2004). At this final alignment stage, the combined transform resulting from motion correction, eddy current correction and anatomical alignment was applied to the raw diffusion data once, and the data were resampled at exactly 2.6 mm isotropic voxels. Next, the table of gradient directions was appropriately adjusted to fit the resampled diffusion data (Leemans and Jones, 2009). Finally, we fitted a tensor model to the diffusion data in each voxel using a standard least-squares algorithm, and extracted the eigenvectors and eigenvalues (λ_1_, λ_2_, λ_3_) of the tensor. Given the single *b*-value used (*b* = 1000), the tensor model is the most appropriate for the analysis of our data. Importantly, at this *b*-value, the tensor model provides high accuracy, similar to more complicated shapes (Rokem et al., 2015). Using the eigenvalues extracted from each tensor, we calculated the FA in each voxel as the weighted standard deviation of the three eigenvalues (Basser and Pierpaoli, 1996). Additional complementary measures were calculated, including axial diffusivity (AD, λ1) and radial diffusivity [RD (λ2+λ3)/2]. AD is defined as the diffusivity along the principal axis of diffusion, and RD as the average diffusivity along the two remaining minor axes. Note that these calculations all took place at the individual patient level in the native space of each patient.

##### Tract identification and segmentation

We focused on a small set of preselected tracts, defined individually in each patient’s native space. Tracts of interest included the fronto-temporal arcuate fasciculus (AF_ft_), frontal aslant tract (FAT), IFOF and uncinate fasciculus (UF), bilaterally. These tracts were selected based on their known involvement in phonological processing (AF_ft_, e.g., Yeatman et al., 2011), semantic processing (IFOF, UF, e.g., Duffau, 2013; Nugiel et al., 2016), or oral fluency (FAT, Catani et al., 2013).

**FIGURE 1:**
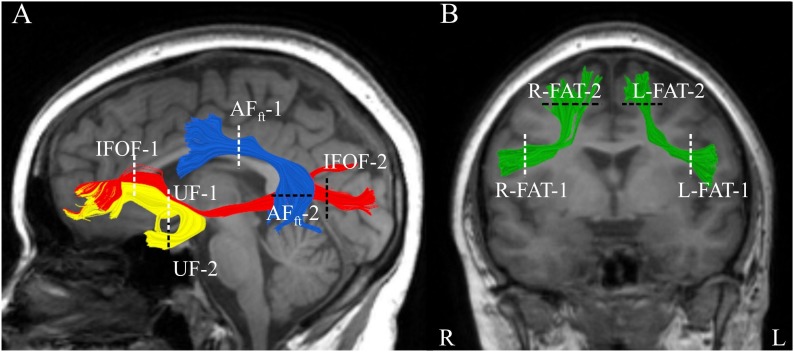
Segmented white matter tracts. Analyzed tracts are visualized in a single patient with MS (Male, 20 years old) overlaid on his T1 images. **(A)** Shown are three left hemisphere tracts: fronto-temporal arcuate fasciculus in blue, uncinate fasciculus in yellow, and inferior fronto-occipital fasciculus in red. **(B)** Left and right frontal aslant tracts are shown in green in the same participant. Dashed lines represent the location of the regions of interest (two for each tract) used to segment the fibers. AF_ft_, fronto-temporal arcuate fasciculus; UF, uncinate fasciculus; IFOF, inferior fronto-occipital fasciculus; FAT, frontal aslant tract.

In order to identify these tracts and quantify their diffusion parameters, we used the Automatic Fiber Quantification (“AFQ”) package, an automated segmentation and quantification tool (Yeatman et al., 2012). AFQ consists of the following steps: (1) Whole brain fiber tractography, (2) Tract segmentation based on region-of-interest (ROI) and automatic cleaning of fiber outliers, and (3) Quantification of diffusion properties along the tracts. For whole brain tracking (step 1), we used deterministic Streamlines Tractography (STT), with a 4th Runge–Kutta path integration method and 1 mm fixed step size (Mori et al., 1999; Basser et al., 2000; Press et al., 2002). Deterministic tractography was used in order to avoid issues pertaining to tract selection (Pestilli et al., 2014) that are not well addressed with a single, relatively low *b*-value scan. Deterministic methods proved to be reliable for the purpose of identifying such large, well-known tracts as the ones identified here (Yeatman et al., 2012). Tract segmentation (step 2) was done in the native space of each patient, using ROIs defined on a T1 template (ICBM, 2009a Non-linear Asymmetric template; Fonov et al., 2011), which were back-transformed into the patient’s native space (see Figure 1 and Supplementary Figure S1 for the definition of all ROIs). Whole brain fibers were restricted to only those that passed through both ROIs, for each tract (following Wakana et al., 2007 for the AF_ft_, IFOF and UF; see Kronfeld-Duenias et al., 2016 for the procedure to segment the FAT using AFQ). After tract segmentation, an automatic cleaning strategy was applied, removing fibers longer than 4 standard deviations from the mean fiber length and those that spatially deviated more than 5 standard deviations from the core of the tract (see Yeatman et al., 2012 for details regarding the automatic segmentation and cleaning procedures).

Quantification of diffusion properties (step 3) was applied as follows: For each participant, for each tract, a FA profile was calculated by sampling 100 equidistant nodes along the core of the tract, between the two ROIs used to segment it. Additionally, a mean tract-FA value was calculated by averaging over all the streamlines within each tract, end to end. The resulting FA-profile and mean tract-FA were subject to further statistical analyses (see section “Brain-Behavior Correlation Analysis” below). Tract profiles provide increased sensitivity to specific clusters of brain-behavior correlations (see, e.g., Travis et al., 2015; Blecher et al., 2016; Kronfeld-Duenias et al., 2016). For tract visualization, we used “Quench,” an interactive tract visualization tool (Akers, 2006).

##### Lesion identification and quantification

MR images were analyzed by an experienced user (SM) to identify MS lesions using in-house developed lesion segmentation software (MS Analyze, MATLAB 7.5). The number and volume of brain lesions were calculated on axial T2 and T2-FLAIR images (slice thickness 3 mm; no gap). Lesions were identified on axial slices and assigned to specific lobes by comparison to a navigated anatomical MRI atlas^2^.

#### Brain-Behavior Correlation Analysis

We used the Kolmogorov–Smirnov test to assess the normality of the data (Corder and Foreman, 2009). Based on the results, Pearson correlation coefficients were calculated between mean-tract FA and each fluency measure, separately. We controlled the false discovery rate (FDR) across the 8 tracts of interest, at *q* = 0.05. Second, for each tract, Pearson correlation coefficients were calculated at each node along the trajectory of the tract. Significance was corrected for 800 correlations using a non-parametric permutation method, yielding a family-wise error (FWE) corrected alpha-value of 0.05 (Nichols and Holmes, 2002). This correction produced an FWE significant cluster size and a corrected alpha value for each tract of interest. We consider a segment significant if (1) the segment includes a cluster of adjacent nodes, each showing a correlation with *p* < 0.05 (uncorrected), and the cluster size is equal or larger than the critical cluster size determined by FWE; or (2) the segment includes any number of nodes that show a correlation with a *P*-value smaller than the corrected alpha determined by FWE. To visualize the pattern of co-variation between FA and fluency scores in significant segments, we extracted the mean FA value within the significant cluster for each patient [in case (2), this was achieved by defining a window of 17 nodes, centered on the most significant node]. Then, we plotted the data from the significant cluster or window against the relevant fluency score. A window size of 17 nodes was selected *a priori* as a reasonable size that balances generality and specificity, but very similar scatter plots were observed with cluster sizes of 13, 15, and 19 (not shown).

Significant correlations along the tract profiles were followed up with multiple regression models and partial correlations. Multiple regression models attempted to explain the variance in each fluency score using as predictors the mean FA in the significant cluster, together with age and education. Partial correlations considered the correlation between fluency and FA while controlling for (one at a time) a variety of demographic, cognitive and clinical factors, including age, gender, education, disease duration, executive function and attention. Executive function and attention were weakly correlated (*r* = 0.38), but we chose to enter them separately into this analysis because they stand for different cognitive components. We applied FDR correction at *q* < 0.05 for those 6 partial correlations. The FA-profile analysis was also followed up with partial correlations controlling for the number of lesions. This procedure resulted in three partial correlations for each significant segment (number of lesions in total, frontal and temporal areas). We applied FDR correction at *q* < 0.05 for those 3 × 2 partial correlations. In addition, significant correlations with one fluency score (letter-based or category-based) were followed up with partial correlations while controlling for the other fluency score.

## Results

The demographic and clinical characteristics of our sample of MS patients (*N* = 33, 11 males, 22 females) are listed in Table 1. Most of the patients (24/33) were diagnosed with relapsing-remitting MS, with very large ranges of disease duration (1–31 years), age (20–62 years), and education (10–22 years) observed across the sample (see Table 1 for detailed EDSS scores, means and standard deviations on each measure).

**Table 1:** Demographics, cognitive and clinical characteristics of the patient sample.

	Mean (SD) [range]^∗^
Age (years)	42.9 (11.88) [20–62]
Males/females	*N* = 11/22
Education (years)	14.15 (2.59) [10–22]
Disease duration (years)	11.21 (6.98) [1–31]
Letter-based fluency (*Z*-scores)	-1.05 (1.06) [(-2.89) to 0.83]
Category-based fluency (*Z*-scores)	-0.74 (1.37) [(-2.93) to 2.24]
**EDSS:**	
Total	4.78 (1.7) [1.5–8]
Pyramidal	2.97 (1.22) [0–5]
Cerebellar	1.64 (1.15) [0–1]
Sensory	1.88 (1.3) [0–4]
**Disease classification:**	
RRMS/total	*N* = 24/33
PPMS/total	*N* = 2/33
SPMS/total	*N* = 7/33


*EDSS, expanded disability status scale; RRMS, relapsing-remitting MS; PPMS, primary progressive MS; SPMS, secondary progressive MS. ^∗^Unless otherwise noted.*

**FIGURE 2:**
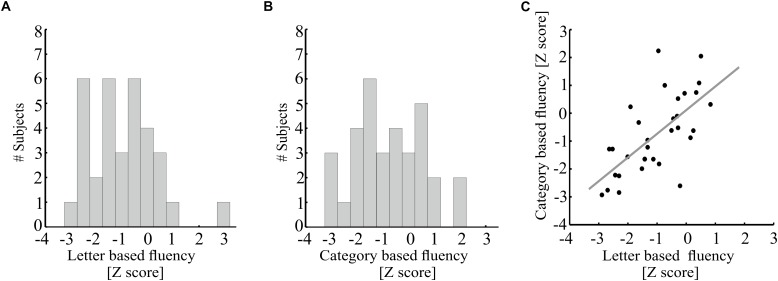
Behavioral performance on the fluency tasks by MS patients. **(A,B)** Distribution of age standardized letter-based **(A)** and category-based **(B)** fluency scores (*N* = 33). **(C)** Age-standardized letter-based fluency scores (*X*-axis) are plotted against age-standardized category-based fluency scores [*N* = 32, one additional participant was excluded from analysis due to a very high letter-based fluency score (*Z* = 2.97)]. A strong positive correlation is found between these two fluency measures (Pearson’s *r* = 0.66, *p* < 0.00005). The gray line represents the best linear fit to these measurements.

Overall, patients with MS obtained lower fluency scores compared to the norms (letter-based: *t*(31) = -5.63, *p* < 0.000005; category-based: *t*(32) = -3.09, *p* < 0.005). Seventy percent of the MS patients in our sample demonstrated below-norm letter- and category-based fluency scores (Figure 2A,B). One patient scored more than 3 standard deviations *above* the norm of letter-based fluency (see Figure 2A) and was therefore considered an outlier and excluded from further analysis. Letter- and category-based fluency scores were strongly correlated (Figure 2C; *r* = 0.66, *p* < 0.00005). This correlation explains the considerable overlap in brain-behavior correlations with each of these fluency components.

The dorsal and ventral tracts of interest were successfully identified bilaterally in almost all of our MS patients (Figure 1), with the exception of 2 patients in whom the bilateral IFOFs were missing, and 1 patient who was missing the right arcuate fasciculus. Supplementary Figures S2–S4 provide visualizations of all segmented tracts in each patient.

**FIGURE 3:**
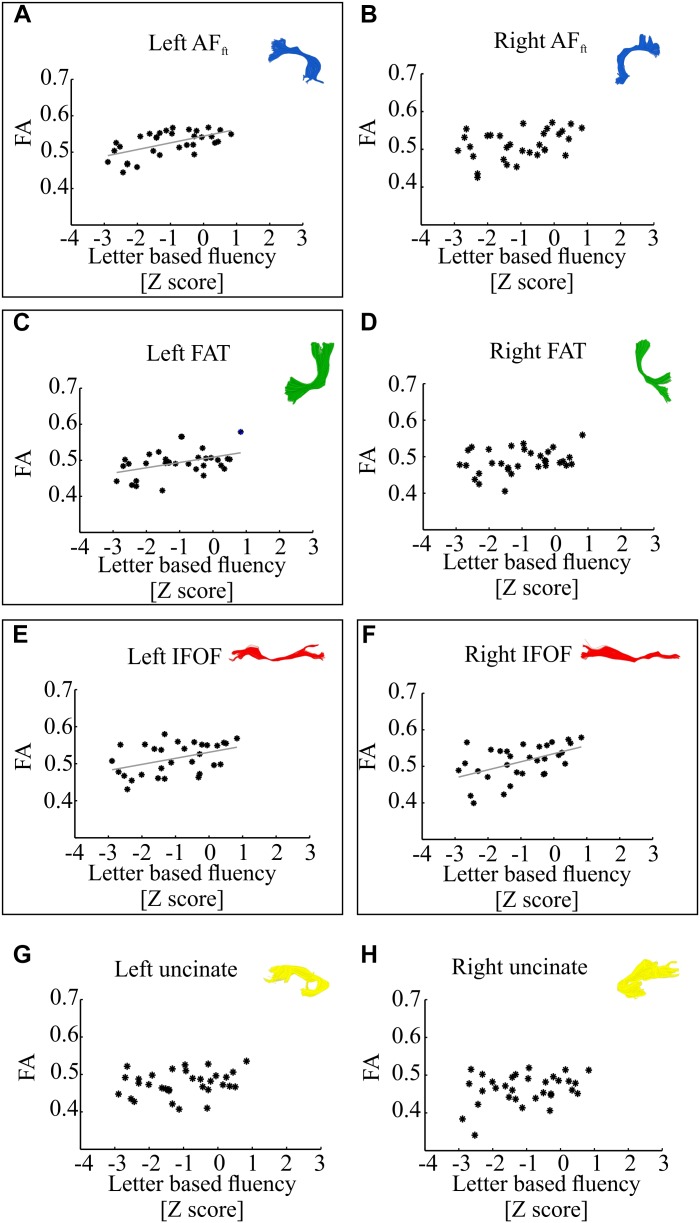
Correlations between letter-based fluency and mean tract-FA. Mean tract-FA is plotted against age-standardized letter-based fluency scores. Each dot represents an individual patient [*N* = 32, one additional participant was excluded from analysis due to a very high letter-based fluency score (*Z* = 2.97)]. Each panel plots these values for a different tract: the left and right fronto-temporal arcuate fasciculus (AF_ft_; **A,B**), frontal aslant tract (FAT; **C,D**), inferior fronto-occipital fasciculus (IFOF; **E,F**), and uncinate fasciculus **(G,H)**. Significant Pearson correlations (FDR corrected for eight comparisons, *q* < 0.05) are framed with a rectangle. Significant correlations were detected in the left AF_ft_ (*r* = 0.58, *p* < 0.001), left FAT (*r* = 0.42, *p* < 0.05), left and right IFOF (*r* = 0.42, *p* < 0.05; *r* = 0.5, *p* < 0.005). Gray lines represent the best linear fits for the significant associations.

**FIGURE 4:**
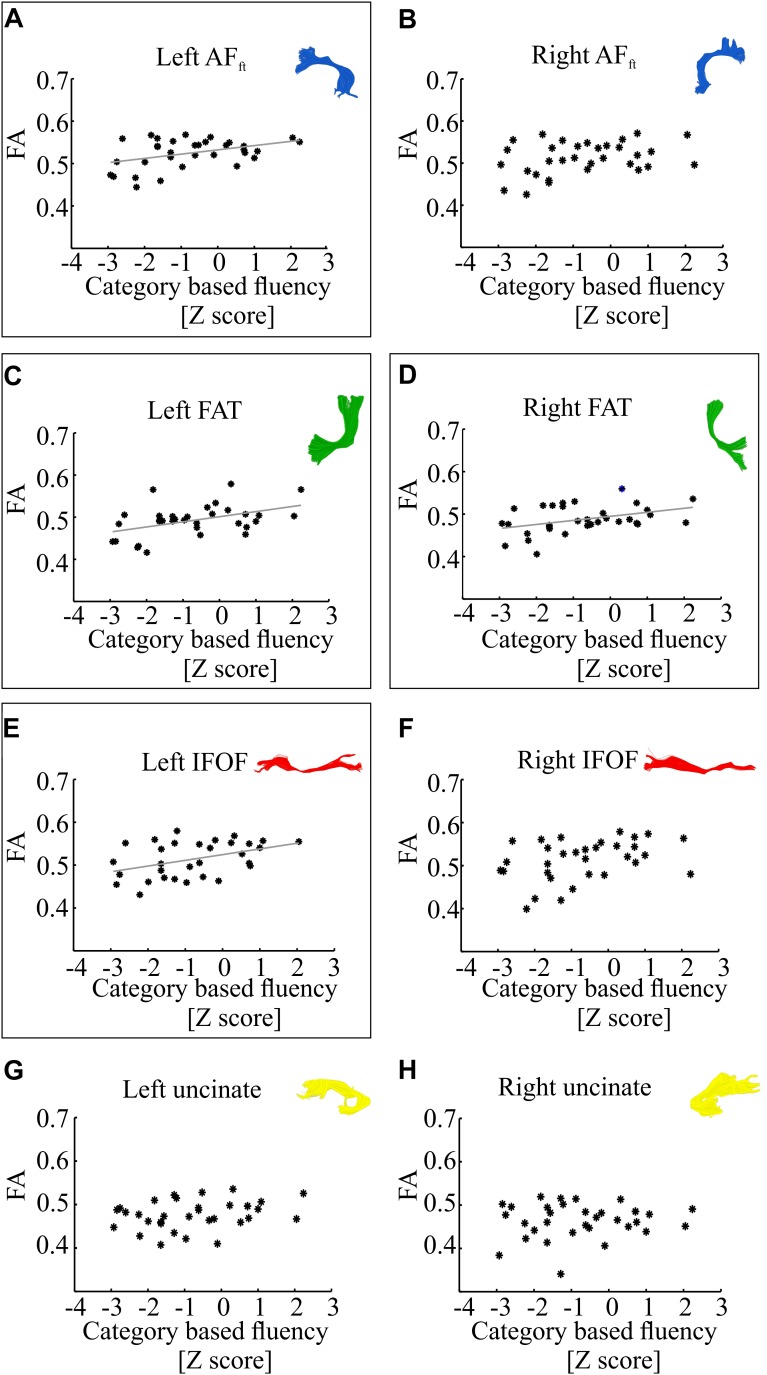
Correlations between category-based fluency and mean tract-FA. Mean tract-FA is plotted against age-standardized category-based fluency scores. Each dot represents an individual patient (*N* = 33). Each panel plots these values for a different tract: the left and right fronto-temporal arcuate fasciculus (AF_ft_; **A,B**), frontal aslant tract (FAT; **C,D**), inferior fronto-occipital fasciculus (IFOF; **E,F**), and uncinate fasciculus **(G,H)**. Significant Pearson correlations (after FDR correction for eight comparisons, *q* < 0.05) are framed with a rectangle. Significant correlations were detected in the left AF_ft_ (*r* = 0.42, *p* < 0.05), left and right FAT (*r* = 0.45, *p* < 0.01; *r* = 0.41, *p* < 0.05), left IFOF (*r* = 0.42, *p* < 0.05). Gray lines represent the best linear fits for the significant associations.

Figure 3 demonstrates correlations between mean tract-FA values and letter-based fluency scores, and Figure 4 shows the same for category-based fluency scores. Black frames indicate significant correlations (corrected for eight comparisons by controlling the FDR at *q* = 0.05). Associations with both fluency components were positive and showed a clear left lateralized trend, across both dorsal (AF_ft_, FAT) and ventral (IFOF) tracts. Significant associations were found with both fluency components in the left AF_ft_, left FAT and left IFOF. Two significant positive associations were detected in right hemispheric tracts: the right IFOF correlated with letter-based fluency and the right FAT was associated with the category-based fluency. No significant association was detected in the left or right UF, for either fluency component.

In order to dissociate between the effects of the two fluency components, we followed up on each of the significant correlation effects by calculating Pearson partial correlations, controlling for the other fluency component. The results showed a strong and significant partial correlation between letter-based fluency and mean FA of the left AF_ft_, when controlling for category-based fluency (see Table 2). These findings help isolate the unique contribution of the left AF_ft_ to lexical access based on a phonemic criterion (opening sound). No significant findings were identified in the complementary analysis, examining partial correlations between mean tract-FA and category-based fluency while controlling for letter-based fluency (Table 3).

**Table 2:** Pearson partial correlations between letter-based fluency and mean tract-FA while controlling for category-based fluency^ϕ^.

White matter tract	Rho	*P*
Left AF_ ft_	0.4513	0.0108^∗^
Left FAT	0.1475	0.4283
Left IFOF	0.1825	0.3435
Right IFOF	0.3788	0.0390


*^ϕ^Follow-up analysis conducted only on the tracts that showed significant correlations in Figure 3 (*N* = 32). ^∗^Significant after controlling FDR for the four significant tracts from Figure 3, *q* < 0.05. Reported are the uncorrected *P*-values.*

**Table 3:** Pearson partial correlations between category-based fluency and mean tract-FA while controlling for letter-based fluency^ϕ^.

White matter tract	Rho	*P*
Left AF_ ft_	0.1347	0.4624
Left FAT	0.3871	0.0286
Right FAT	0.3488	0.0504
Left IFOF	0.2843	0.1278


*^ϕ^Follow-up analysis conducted only on the tracts that showed significant correlations in Figure 4 (*N* = 33). None of the partial correlations were significant when controlling FDR for the four significant tracts from Figure 4 at *q* < 0.05. Reported are the uncorrected *P*-values.*

**FIGURE 5:**
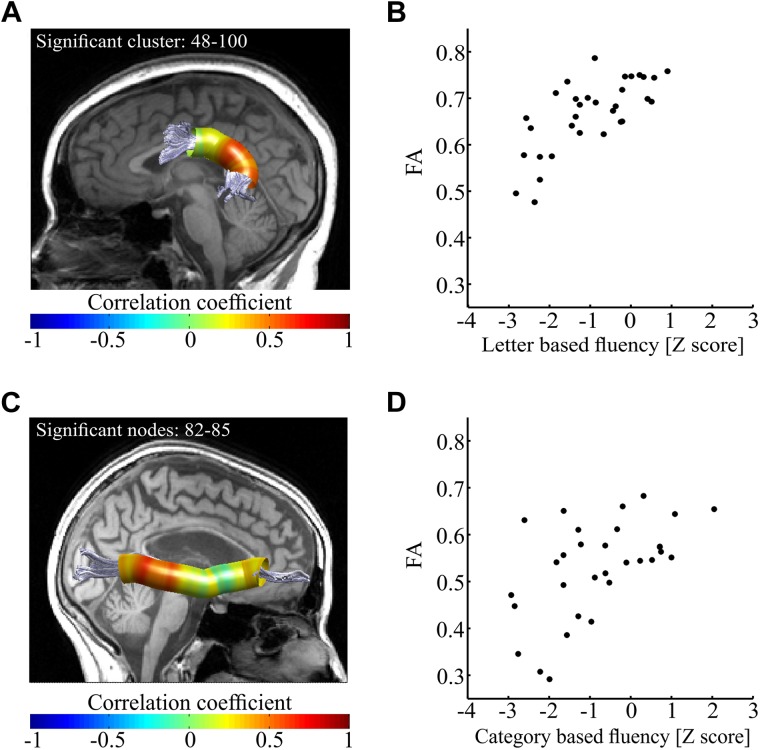
Correlations between fluency components and FA along the tract. **(A,B)** Correlations between letter-based fluency and FA along the left AF_ft_. Panel **(A)** shows the left AF_ft_ in a single participant. Colored overlay represents Pearson’s *r*-values between letter-based fluency scores and FA values along the core of the tract (*N* = 32). Significant correlations were detected in a posterior cluster of 53 nodes (*p* < 0.05, FWE corrected for 800 comparisons, critical *r* = 0.599). In panel **(B)**, mean FA of the significant cluster (nodes 48–100) is plotted against the letter-based fluency scores for each participant. This depiction is provided for visualization purposes only. **(C,D)** Correlations between category-based fluency and FA along the right IFOF. Panel **(C)** shows the right IFOF in a single participant. Colored overlay represents Pearson’s *r*-values between category-based fluency scores and FA values along the core of the tract (*N* = 31, 2 additional participants were excluded from analysis because we could not identify the R-IFOF in their data). Significant correlations were detected in a posterior cluster of four nodes (*p* < 0.05, FWE corrected for 800 comparisons, critical *r* = 0.611). In panel **(B)**, mean FA of a window of 17 nodes around the most significant node (#83) is plotted against the category-based fluency scores for each participant. Here too, this depiction is provided for visualization purposes only. AF_ft_, fronto-temporal arcuate fasciculus; IFOF, inferior fronto-occipital fasciculus.

Having detected a broad pattern of correlations between fluency and mean tract-FA, we next attempted to detect the specific segments within the tracts of interest that drive these correlations. In order to do so, we extracted FA in 100 equi-spaced nodes along the core of the tract (see section “Materials and Methods”), and calculated Pearson correlations between each fluency measure and FA along the tract, node by node. This analysis exposed a divergent pattern of results for letter-based and category based fluency. Letter-based fluency was significantly and positively correlated with FA in a large cluster of 53 nodes along the left AF_ft_ (*p* < 0.05, FWE corrected for 800 nodes, critical *r* = 0.599, see Figure 5A,B). This correlation remained significant even after partialling out the effect of category-based fluency (*r* = 0.525, *p* < 0.005). It also remained significant after controlling for various demographic variables, including age, gender, education, disease duration, executive function and attention (see Supplementary Table S2).

**FIGURE 6:**
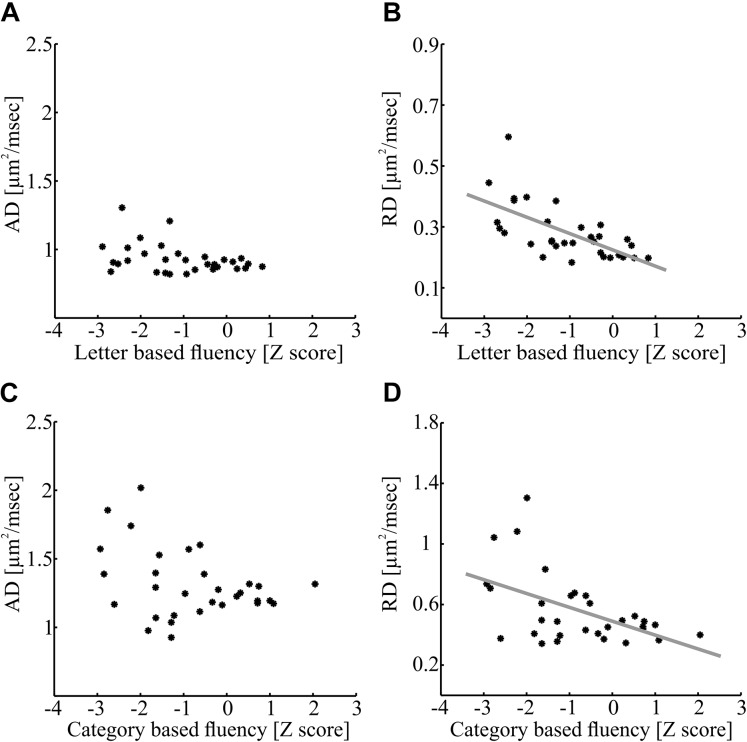
Axial diffusivity and RD correlations with fluency measures within significant clusters. **(A,B)** Correlations between letter-based fluency and diffusivity measures along the left AF_ft_. Mean AD **(A)** and RD **(B)** values of the significant cluster depicted in Figure 5A (nodes 48–100) are plotted against the letter-based fluency scores for each participant (*N* = 32; **A:** *p* > 0.05; **B:** *p* < 0.0001). **(C,D)** Correlations between category-based fluency and diffusivity measures along the right IFOF. Mean AD **(C)** and RD **(D)** of the significant cluster depicted in Figure 5C (nodes 75–91) is plotted against the category-based fluency scores for each participant (*N* = 31; **C:** *p* > 0.05; **D:** *p* < 0.005). AF_ft_, fronto-temporal arcuate fasciculus; IFOF, inferior fronto-occipital fasciculus.

In contrast with these findings, category-based fluency was significantly and positively correlated with FA in a small cluster within the right IFOF (*p* < 0.05, FWE corrected for 800 nodes, critical *r* = 0.611, see Figure 5C,D). This correlation, too, remained significant after controlling for various demographic variables (see Supplementary Table S3). We followed up on the significant correlations with *post hoc* correlations replacing FA with mean AD and mean RD of the significant segments. We found a significant correlation between letter-based fluency and RD (*r* = -0.64, *p* < 0.0001), while the correlation with AD was not significant (Figure 6A,B). The same pattern was found for category-based fluency (Figure 6C,D). No significant correlations were detected in the profile analyses along the left and right FAT, left and right UF, left IFOF and right AF_ft_.

In another follow up analysis, we assessed the explanatory power of FA in the significant cluster by calculating multiple regression models, which predicted each fluency component based on FA in the significant cluster, considering two additional demographic variables: age and education. The results of these analyses are presented in Tables 4, 5, and demonstrate, once again, a solid effect of FA in the left AF_ft_ in explaining letter-based fluency (the full model explained 58% of the variance in letter-based fluency, with a significant effect of FA but non-significant contributions of age and education). Consistent with the mean tract analysis, the results in the R-IFOF were not as convincing: a significant effect of age was observed, washing away the effect of FA, and the model as a whole explained 38% of the variance in category-based fluency scores.

**Table 4:** Results of a multiple regression analysis predicting letter-based fluency scores, based on FA in the significant cluster of the left fronto-temporal arcuate fasciculus, as well as participants’ age and education.

	*t*	*P*	SE
Mean FA of L AF (significant cluster)	-4.35	2.14e^-4∗^	-2.03
Age	0.96	0.34	-0.01
Education	-1.94	0.06	-0.05


*N = 32; the full model explains 58% of the variance in letter-based fluency scores. ^∗^*P* < 0.05.*

**Table 5:** Results of a multiple regression analysis predicting category-based fluency scores, based on FA in the significant window of the right inferior fronto-occipital fasciculus, as well as participants’ age and education.

	*t*	*P*	SE
Mean FA of R IFOF (significant cluster)	-0.33	0.74	-2.26
Age	-2.47	0.02^∗^	-0.02
Education	-1.86	0.07	-0.84


*N = 31; the full model explains 38% of the variance in category-based fluency scores. ^∗^*P* < 0.05.*

Finally, we assessed the effect of identified MS lesions on the microstructural measures and correlations observed in our sample. To this end, we first examined correlations between mean tract-FA values and the number of identified lesions in each lobe. Indeed, significant correlations were identified between tract-FA of the AF_ft_ and FAT and the total number of lesions, as well as the number of frontal and temporal lobe lesions. In contrast, no significant correlations were found between ventral stream tract-FA measures and the number of lesions anywhere in the brain (see Supplementary Table S4). Based on these findings, particularly the correlations found in dorsal tracts with number of lesions, we re-examined the correlations between FA in specific tract segments and fluency components (Figure 5). In both significant segments (i.e., in the left AF_ft_ and right IFOF), partial correlations between FA and fluency remained significant after controlling for the total number of lesions, frontal or temporal lobe lesions (Table 6). This finding suggests that the specific correlations observed in Figure 5 reflect variation in microstructural properties rather than clinically identified lesions.

**Table 6:** Pearson partial correlations between mean FA of a significant segment and verbal fluency, while controlling for number of lesions located in the frontal, temporal, and total areas^ϕ^.

Controlled variable	Left AF_ft_ – letter-based fluency		Right IFOF – category-based fluency	
		
	Rho	*P*	Rho	*P*
Total	0.718	0.00544 × 10^-3∗^	0.6252	0.2208 × 10^-3∗^
Frontal	0.7089	0.00805 × 10^-3∗^	0.6275	0.2062 × 10^-3∗^
Temporal	0.7191	0.00517 × 10^-3∗^	0.6337	0.1703 × 10^-3∗^


*^ϕ^Follow-up analysis conducted only on the tracts that showed significant correlations in Figure 5. ^∗^Significant correlations after controlling FDR for six comparisons, *q* < 0.05. Reported are the uncorrected *P*-values.*

## Discussion

In this study, we assessed the association between verbal fluency and microstructural properties of white matter pathways in MS patients. We hypothesized that verbal fluency will be correlated with FA in the left dorsal and bilateral ventral language pathways. Indeed, our findings support this hypothesis: a whole-tract analysis revealed significant correlations between verbal fluency and FA of the left AF_ft_ and left FAT (dorsal) as well as with FA of the IFOF (ventral), bilaterally. The analysis of FA-profiles along the tracts revealed a more selective correlation pattern. We found a significant correlation between letter-based fluency and FA in a large segment of the left AF_ft_ (dorsal stream), while category-based fluency correlated with FA in a posterior segment of the right IFOF (ventral stream). These correlations remained significant even when controlling for the other fluency parameter, demonstrating some level of dissociation between the white matter pathways supporting different fluency components.

Left lateralization of the language systems in the brain is a well-established concept (Catani et al., 2005; Friederici et al., 2006; Vernooij et al., 2007). Indeed, our analysis of mean tract-FA correlations with verbal fluency revealed a left lateralized, broad distribution of correlations, highlighting the left arcuate, FAT and IFOF, across both fluency components. The distributed pattern of correlations detected in this analysis, across both dorsal and ventral pathways, could be attributed to the nature of the fluency task. This task engages multiple aspects of language processing: comprehension (of the cue), lexical search according to a predefined criterion, lexical access, speech programming and fluent production. It also probes the interface between language and other cognitive domains, such as executive functions, specifically cognitive control and working memory (switching quickly from one subcategory to another, keeping track of prior responses and avoiding repetitions are all essential for high fluency scores). This comprehensive nature is exactly what makes verbal fluency a popular clinical task, because it can be used to detect many aspects of cognitive failure. Consistent with this multi-componential nature, verbal fluency also gives rise to a broad pattern of correlations across multiple language-related pathways.

The dissociation we detect between letter-based and category-based fluency fits naturally within the dual stream model of speech processing (Hickok and Poeppel, 2007; Saur et al., 2008; Poeppel, 2014). Our results showed that segments of the left AF_ft_ and of the right IFOF were correlated with letter-based fluency and category-based fluency, respectively. The fronto-temporal branch of the AF, which is the branch analyzed here, is well known as a left-dominant language pathway (Catani et al., 2005; Catani and Mesulam, 2008; Thiebaut de Schotten et al., 2011). This pathway is consistently (but not uniquely) correlated with measures of phonological processing (Yeatman et al., 2011; Saygin et al., 2013; Vanderauwera et al., 2015). The partial correlation observed in the left AF_ft_ with letter-based fluency (while controlling for category-based fluency) fits with the idea that this subtest, often labeled phonemic fluency, indeed highlights phonological aspects of lexical search, over and above the shared cognitive processes engaged in both subtests. The IFOF, on the other hand, is a bilateral or right-lateralized tract (Thiebaut de Schotten et al., 2011), and has been associated with semantic processing and lexical access (Saur et al., 2008; Cummine et al., 2015; Harvey and Schnur, 2015; Nugiel et al., 2016). The selective correlation pattern detected in our FA profile analysis thus provides additional support from a new clinical population to the dual stream model of language processing.

Follow up analyses further supported the added value of tract properties in explaining fluency performance, over and above demographic and cognitive measures. Specifically, using multiple regression models, we found that FA of the significant cluster in the left AF_ft_ significantly predicts letter-based fluency. Together with (non-significant) contributions of age and education, this model explained 58% of the variance in letter-based fluency. The correlations in both tracts survived partialling out of various demographic, cognitive and clinical measures (Supplementary Tables S2, S3). However, age surpassed the contribution of the right IFOF in explaining category-based fluency, in a regression model that explained, in total, only 38% of the variance. Thus, the effect of the R-IFOF should be interpreted with caution. The minimal effect of disease duration in explaining fluency correlations in MS patients suggests that the reported correlations do not reflect the clinical state. Instead, MS may have enhanced individual variability in white matter microstructural measures, thus exposing neurocognitive correlations that may exist in the healthy population as well.

Previous studies of verbal fluency have shown differences in cortical activation and structure between clinical populations and healthy ones. Specifically, these studies showed patterns of reduced left lateralization when verbal fluency tasks were performed by patients with Alzheimer’s disease, epilepsy and schizophrenia (Weiss et al., 2004; Metternich et al., 2014; Rodríguez-Aranda et al., 2016). A recent study, focusing primarily on the limbic system in MS patients, showed associations between verbal fluency and the UF, bilaterally, and in particular, an association between category-based fluency and the right UF (Keser et al., 2018). The correlation we found in the right IFOF, a neighboring right hemispheric ventral pathway, aligns with this overall tendency for reduced left lateralization in clinical populations. It is still unclear, however, if this correlation reflects the age and clinical status of our population, or whether this is part of the brain’s reorganization for the purpose of compensation. For example, in low-grade Glioma patients with left hemispheric lesions, damage to the left IFOF has been associated with semantic fluency performance (Almairac et al., 2015; see also Li et al., 2017 for a similar association in stroke patients). Further research in both patients and controls will help clarify if our finding in the right IFOF generalizes to the healthy population. If this finding is not replicated in healthy controls, longitudinal studies in MS patients will be necessary to elucidate the temporal evolution of this correlation, which will help determine the causal or compensatory role of the right IFOF in this clinical population.

Prior voxel-based lesion-symptom (VLSM) studies have shown that letter- and category-based fluency are associated with frontal and temporal stroke lesions, respectively (Baldo et al., 2006; Biesbroek et al., 2016). Using pathway-based analysis in MS patients, we did not identify a significant association between the number of frontal or temporal MS lesions and verbal fluency scores. We did find negative correlations between the total number of lesions (nor between the number of frontal or temporal lesions) and mean FA of the left AF_ft_ and left FAT (see Supplementary Table S4). The current study, therefore, suggests that the association between the location of the lesions and the deficit in verbal fluency may be indirect. Lesions in MS patients have an impact on the whole tract and not only on the white matter in the lesions’ location (Droby et al., 2015). The number of lesions does not reflect the severity of the damage to white matter, hence pathway-based analysis provides a more sensitive measure than lesion count.

*Post hoc* analyses revealed that significant FA correlations along the tract were driven by RD and not by AD (see Figure 6). Larger RD levels were associated in our data with lower verbal fluency scores. RD is affected by several factors, including fiber density, myelination, axonal diameter, axonal swelling, and directional coherence (Alexander et al., 2007; Jones et al., 2013). Low fiber density or low amount of myelin allow faster diffusivity of extracellular water molecules in the radial direction leading to elevated RD values (Song et al., 2002). Additionally, thicker axons and axonal swelling allow faster diffusivity in the radial direction of intracellular water molecules (Alexander et al., 2007; Horowitz et al., 2014). It had been suggested that RD is related to demyelination in MS patients (Klawiter et al., 2011). Axonal swelling can also affect our results, due to the inflammatory nature of the disease. On the other hand, it is less likely that directional coherence influenced the correlations since they were found in large clusters of the AF_ft_ and IFOF that are not co-localized with known divergence points of these tracts (see Yeatman et al., 2011). Given that there was no significant correlation with AD, it is unlikely that the results are driven by axonal injuries. Based on these arguments, we explain the reduction of fluency abilities in some MS patients as a result of demyelination, axonal swelling and a decrease in fiber density along specific pathways involved in phonological and semantic processing, lexical access and articulation.

The current study has several limitations. First, our sample includes only MS patients, without a control group of healthy participants, because the data were collected as part of the clinical follow-up of MS patients. No available data exist using the same scan protocol in a matched sample of healthy individuals. While this precludes a group comparison approach, this dataset still allows an individual difference correlational approach, which is the one we take here. In fact, given the considerable variability in severity, age and other biographic measures, we consider an individual difference approach more productive in this sample, and it is quite likely that group differences would be masked by this variability. Importantly, it should be acknowledged that the findings may not directly generalize to the typical adult population. A second limitation concerns potential confounding effects: Because this is a retrospective study, there is a large number of demographic and clinical variables that vary dramatically across the sample. We addressed this issue by calculating partial correlations, controlling for various demographic, cognitive and structural variables. Future prospective studies, including age and education matched control groups, can provide tighter ranges of age and severity and allow better control over these factors. Lastly, while FA provides a measure of white matter microstructure, it is affected by several factors that are hard to disentangle. The fact that the results were driven by RD highlights the potential contribution of reduced fiber density, reduced myelin content or inflammation to the impairment in verbal fluency. Future studies may take advantage of new quantitative imaging techniques in order to probe more directly the macromolecular content of white matter (Assaf et al., 2008; Stikov et al., 2011; Mezer et al., 2013).

## Conclusion

In conclusion, this study revealed several interesting results. On the one hand, it showed that verbal fluency is positively correlated with the mean FA of dorsal and ventral tracts, mostly in the left hemisphere of MS patients. This finding suggests that verbal fluency relies on multiple pathways, potentially contributing to the multiple cognitive aspects probed by this complex task. On the other hand, we identified a potential dissociation between letter-based and category-based fluency, each highlighting a different stream of language processing, dorsal and ventral, respectively. Much like primary progressive aphasia, MS leads to extended variability in white matter properties and in cognitive abilities, thus exposing subtle patterns of neurocognitive correlations that may not have been identified in adult healthy individuals. Extending the range of cognitive neuroscience studies in MS patients will contribute to our understanding of the extent and nature of cognitive impairments in this disease, and contribute to building a reliable model of the functional neuroanatomy underlying complex cognitive processes in the adult human brain.

## Ethics Statement

The data were collected as part of the routine follow-up of the patients. Participants signed a written informed consent according to protocols approved by the ethics committee of the Sheba Medical Center, in accordance with the Declaration of Helsinki. Participants indicated that they agree that their data may be used anonymously for research purposes.

## Author Contributions

TB, MB-S, AA, and SM initiated and planned the study. GS collected and analyzed the behavioral data. SM collected the imaging data. TB and SM analyzed the imaging data. TB conducted the statistical analyses. All authors took part in writing the manuscript.

## Conflict of Interest Statement

The authors declare that the research was conducted in the absence of any commercial or financial relationships that could be construed as a potential conflict of interest.

## Footnotes

1 http://web.stanford.edu/group/vista/cgi-bin/wiki/index.php/MrDiffusion

2 http://headneckbrainspine.com
