# Supplementary material for: Association Between White Matter Microstructure and Verbal Fluency in Patients With Multiple Sclerosis
Source: Front Psychol. 2019 Jul 18;10:1607. doi: 10.3389/fpsyg.2019.01607 (PMC6657651; doi:10.3389/fpsyg.2019.01607)
Supplement: Supplementary file 1 [file Data_Sheet_1.pdf]

Supplementary materials

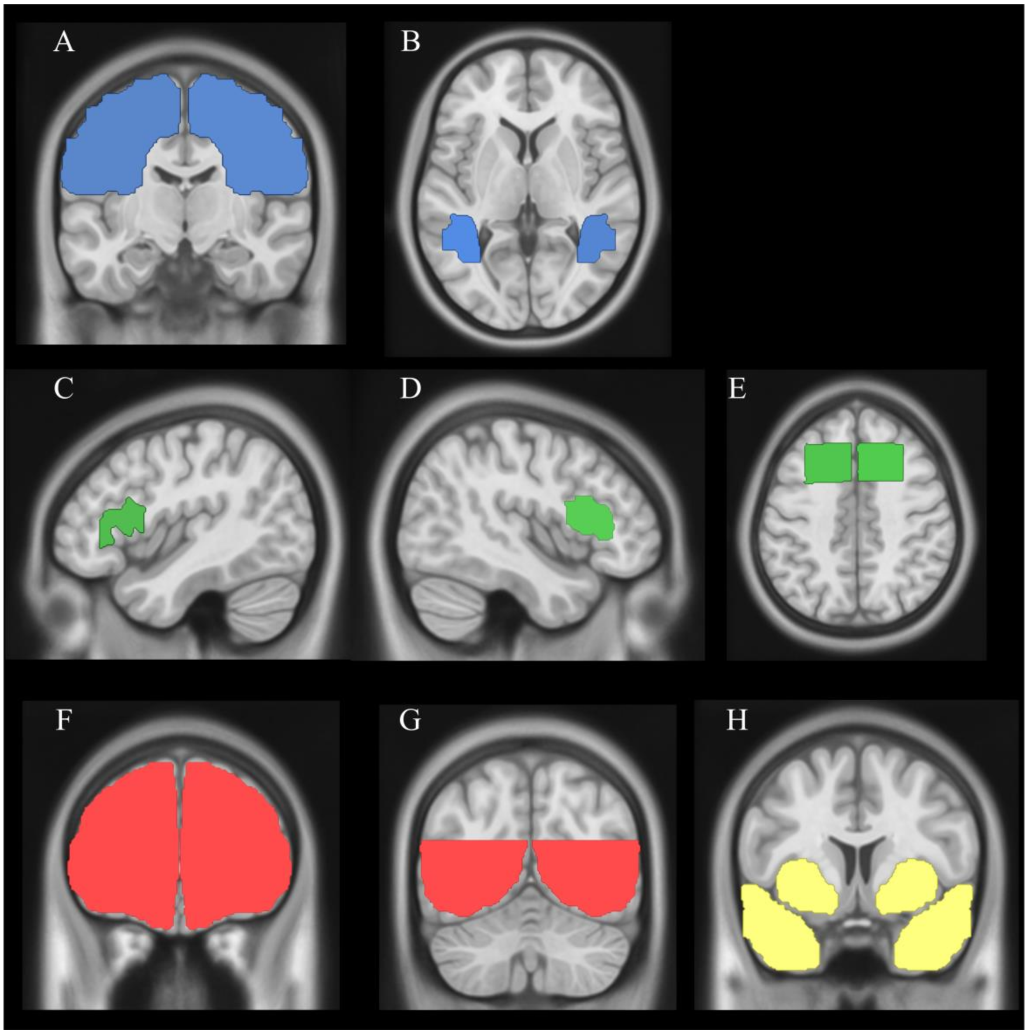

Supplementary Figure 1. ROIs used for tract segmentation. ROIs are visualized on the MNI 152 T1 template, later warped to each patient's brain (see Methods). A) Left and right AF_{ft}-1 ROIs (Figure 1. For more details see Yeatman et al., 2011). B) Left and right AF_{ft}-2 ROIs. C) Left FAT-1 ROI . D) Right FAT-1 ROI . E) Left and right FAT-2 ROIs. F) Left and right IFOF-1 ROIs. G) Left and right IFOF-2 ROIs. H) Left and right UF-1 ROIs (dorsal), left and right UF-2 ROIs (ventral). AF_{ft} = fronto-temporal arcuate fasciculus (blue), FAT = frontal aslant tract (green), IFOF = inferior fronto-occipital fasciculus (red), UF = uncinate fasciculus (yellow).

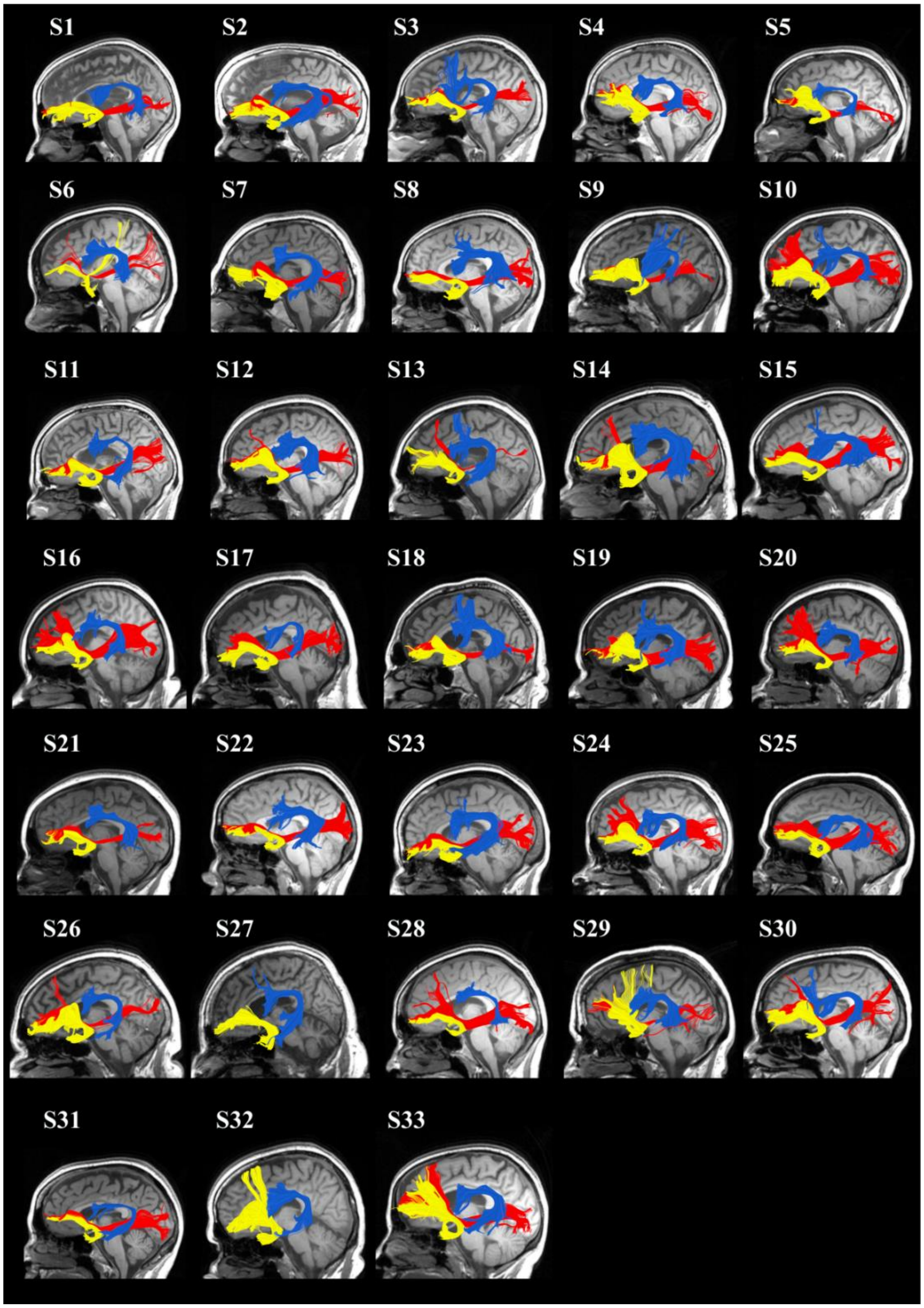

Supplementary Figure 2. Individual segmentations of the left IFOF, UF and AF$_{ft}$. Tracts are overlaid on a mid-sagittal T1 image of each patient (N=33). AF$_{ft}$ = fronto-temporal arcuate fasciculus (blue), IFOF = inferior fronto-occipital fasciculus (red), UF = uncinate fasciculus (yellow).

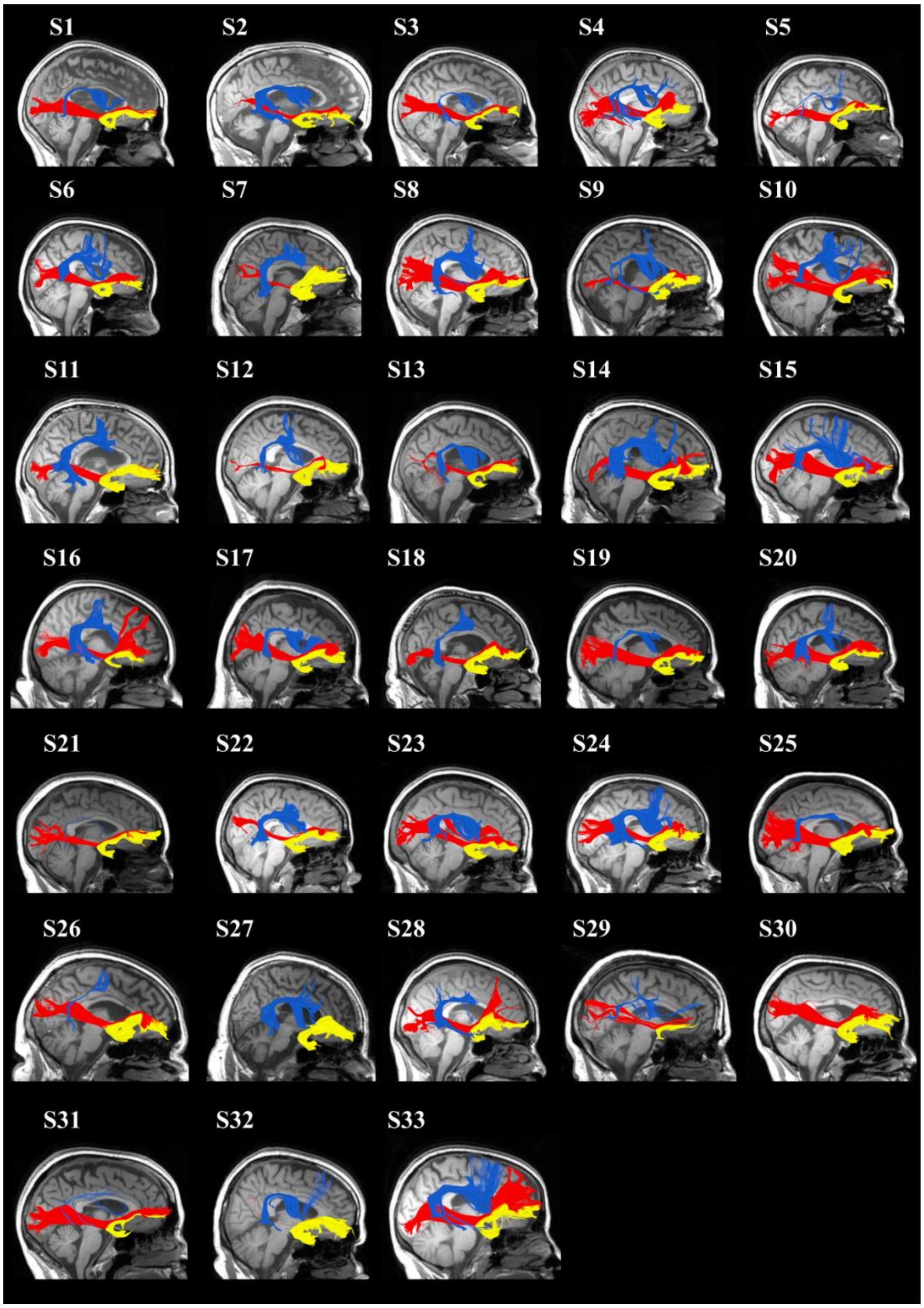

Supplementary Figure 3. Individual segmentations of the right IFOF, UF and AF_{ft}. Tracts are overlaid on a mid-sagittal T1 image of each patient (N=33). AF_{ft} = fronto-temporal arcuate fasciculus (blue), IFOF = inferior fronto-occipital fasciculus (red), UF = uncinate fasciculus (yellow).

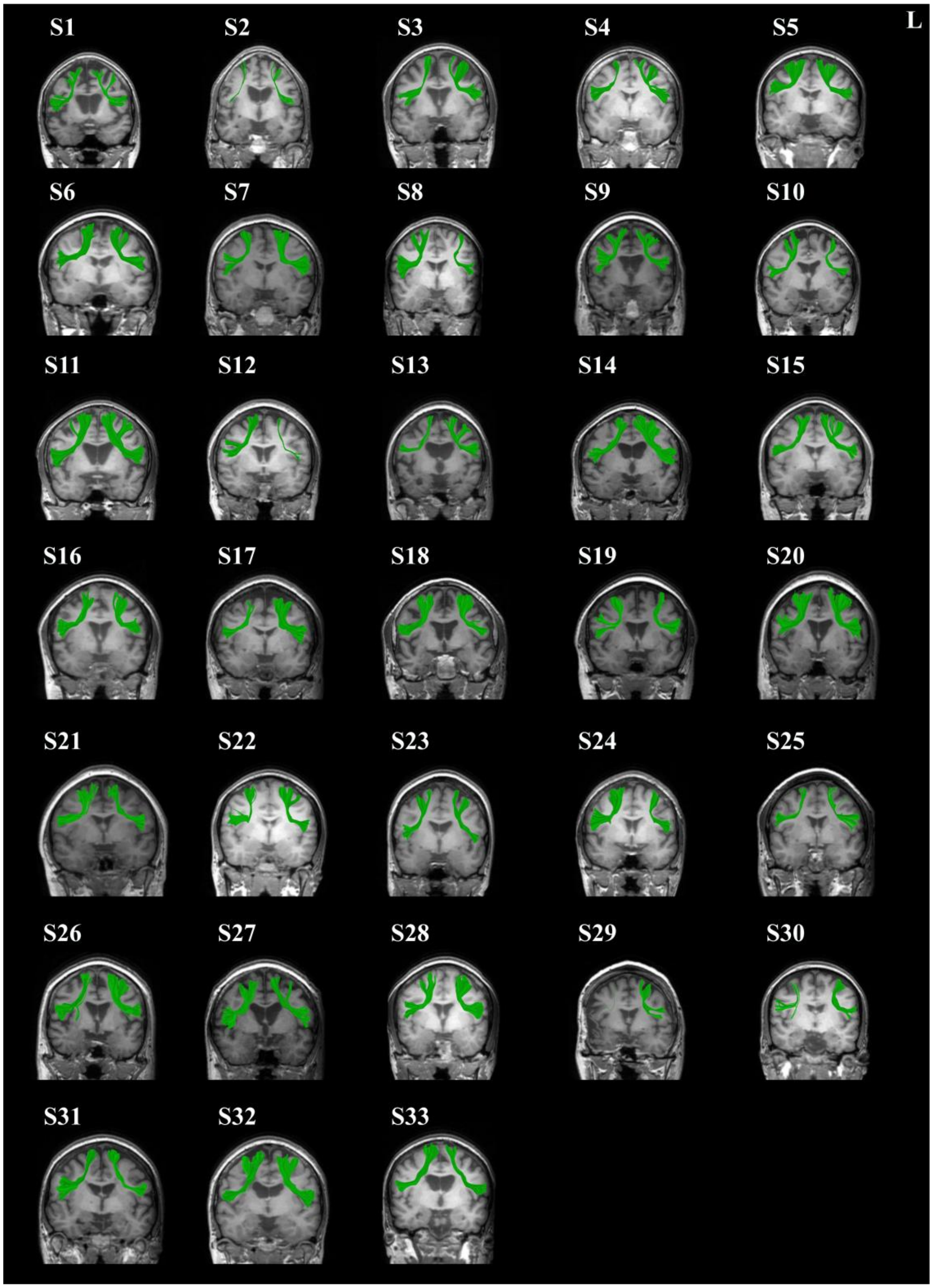

Supplementary Figure 4. Individual segmentations of the bilateral frontal aslant tract (FAT). Segmentations are overlaid on a coronal T1 image for each patient (N=33).

Supplementary Table S1

Demographics and fluency measures of the superset sample (N=78) and the current sample (N=33), demonstrating that the inclusion criterion did not bias our sample in any systematic way.

	Superset sample (N=78) Mean (SD) [range]	Current sample (N=33) Mean (SD) [range]
Age	44 (12) [20 – 64]	42.9 (11.8) [20 – 62]
Education (years)	14.3 (2.68) [10 – 24]	14.15 (2.59) [10 – 22]
Disease duration	12.4 (8.4) [1 – 43]	11.2 (6.98) [1 – 31]
Category-based fluency (Z-scores)	-0.81 (1.38) [-3.87 – 4.42]	-0.74 (1.37) [-2.93 – 2.24]
Letter-based fluency (Z-scores)	-0.98 (1.3) [-3.09 – 2.98]	-1.05 (1.06) [-2.89 – 0.83]

*No significant differences were observed between the groups in any of these measures (p>0.1).

Supplementary Table S2

Pearson partial correlations between letter-based fluency and mean FA of the significant cluster in the left AF_{ft} (See Figure 5A,B), while controlling for various demographic or cognitive variables

Controlled variable	Rho	P
Age	0.7092	0.0080×10^{-3}*
Gender	0.7201	0.0050×10^{-3}*
Education	0.6359	0.1208×10^{-3}*
Disease Duration	0.7178	0.0055×10^{-3}*
Executive function	0.7162	0.0059×10^{-3}*
Attention	0.7117	0.0072×10^{-3}*

N=32.
Significant cluster found within the left fronto-temporal arcuate fasciculus (AF_{ft}).
*Significant after controlling the FDR at q<0.05 for 6 partial correlations

Supplementary Table S3

Pearson partial correlations between category-based fluency and mean FA of the significant window in the right IFOF (See Figure 5C,D), while controlling for various demographic or cognitive variables

Controlled variable	Rho	P
Age	0.5257	0.0029*
Gender	0.5231	0.0030*
Education	0.4555	0.0114*
Disease Duration	0.6076	0.0003*
Executive function	0.556	0.0014*
Attention	0.543	0.0019*

N=31.
Significant window found within the right inferior fronto-occipital fasciculus (IFOF).
*Significant after controlling the FDR at q<0.05 for 6 partial correlations

Supplementary Table S4

Pearson correlations between mean tract-FA and number of lesions in different brain areas.

Lesions location	Left AF_{ft}		Right AF_{ft}		Left FAT		Right FAT		Left IFOF		Right IFOF		Left Uncinate		Right Uncinate	
	Rho	P	Rho	P	Rho	P	Rho	P	Rho	P	Rho	P	Rho	P	Rho	P
Total	-0.4659	0.0063*	-0.4202	0.0166	-0.4923	0.0036*	-0.3649	0.0368	-0.03586	0.0476	-0.4006	0.0231	-0.1861	0.2997	-0.2136	0.2327
Frontal	-0.5102	0.0024*	-0.4475	0.0102	-0.5378	0.0012*	-0.4373	0.0109	-0.3827	0.0336	-0.4565	0.0086	-0.2022	0.2592	-0.2397	0.1792
Temporal	-0.4801	0.0047*	-0.4845	0.005*	-0.5084	0.0025*	-0.3759	0.0311	-0.3576	0.0483	-0.3735	0.0353	-0.1246	0.4897	-0.1187	0.5106
Parietal	-0.3641	0.0372	-0.276	0.1262	-0.3574	0.0411	-0.2294	0.199	-0.3153	0.0841	-0.3043	0.0904	-0.1505	0.4032	-0.1575	0.3814
Occipital	-0.3661	0.0361	-0.3829	0.0305	-0.3721	0.033	-0.2438	0.1715	-0.2976	0.104	-0.3023	0.0926	-0.0813	0.6529	-0.1865	0.2988
Sotto Tentoriale	-0.2097	0.2415	-0.271	0.1336	-0.2485	0.1632	-0.1425	0.4289	-0.0891	0.6336	-0.1665	0.3625	-0.261	0.1423	-0.2937	0.0972

N=33.

*Significant after controlling FDR for 8 fibers×6 parameters, q<0.05.
